# Taping-induced cutaneous stimulation to the ankle tendons reduces minimum toe clearance variability

**DOI:** 10.1016/j.heliyon.2022.e12682

**Published:** 2023-01-06

**Authors:** Prabhat Pathak, Jooeun Ahn

**Affiliations:** a John A. Paulson School of Engineering and Applied Sciences, Harvard University, Cambridge, MA, USA; b Department of Physical Education, Seoul National University, Seoul, Republic of Korea; c Institute of Sport Science, Seoul National University, Seoul, Republic of Korea

**Keywords:** Minimum toe clearance, Cutaneous stimulation, Elastic adhesive tape

## Abstract

Large variability of minimum toe clearance (MTC) leads to a higher risk of tripping. Visual feedback-based gait training systems have been used to regulate MTC distribution, but these systems are expensive and bulky. Furthermore, the effect of such training lasts only for a short period of time. Considering the efficacy of elastic adhesive tape-induced cutaneous stimulation to the ankle tendons in improving proprioception and movement detection, we hypothesize that application of tapes to the ankle tendons as a practical method for modifying MTC distribution. To test this hypothesis, we recruited 13 young and healthy adults and instructed them to walk on a treadmill under four conditions: no taping, taping the tibialis anterior tendon, taping the Achilles tendon, and taping both tendons. We measured MTC distribution, lower limb joint angles and muscle activations of the tibialis anterior and gastrocnemius medialis, and compared these outcomes under the four conditions. The application of elastic adhesive tape to the ankle tendons had no significant effect on the average MTC height, but tapes applied to the Achilles tendon and both tendons significantly reduced MTC variability. Taping decreased the variability of some lower limb joint angles, but taping did not induce significant changes in the activation levels of the shank muscles. These results demonstrate that elastic adhesive tape applied to the shank can reduce MTC variability with minimal resistance, inertia and cumbersomeness.

## Highlights

• Large variability of minimum toe clearance (MTC) increases the risk of tripping.
• Cutaneous stimulation can enhance proprioception and control of the foot.
• Elastic adhesive tape can provide cutaneous stimulation without discomfort.
• We demonstrated that taping ankle tendons actually reduces MTC variability.

## 1 Introduction

Reliable control of foot trajectory is strongly associated with stable walking [1,2]. In particular, the smallest distance from the ground to the toe during swing phase, or the minimum toe clearance (MTC) is directly related with the risk of accidental contact with the ground and tripping-related falls [3,4]. Accordingly, the distribution of MTC has been used as a salient indicator of the risk of tripping; the elderly and patients with neuromuscular diseases show decreased mean MTC height and increased MTC variability [[5], [6], [7]].

A limited number of studies explored methods for modifying this MTC distribution. Using a system that provides real-time visual feedback on toe clearance, Tirosh et al. trained healthy young adults to increase the MTC height [8], and Begg et al. trained older adults and individuals with stroke to reduce MTC variability [9]. However, this training system, which requires a large screen, treadmill, and motion tracking device is bulky and expensive. More importantly, the practicality of this training system is hampered by the limited duration of the beneficial effect; the retention period of the changes in MTC distribution is only similar to the training period.

Exploiting the effect of tactile sensation on motor variability, recent studies suggested more compact and practical methods for modifying MTC distribution. Yamashita et al. showed that cutaneous stimulation to the soles of feet via mechanical vibration reduces the variability of toe trajectory during swing phase [10], and Pathak et al. demonstrated that supra-threshold tactile stimulation applied to soles through vibrating insoles can reduce MTC variability [11]. However, proper function of these interventions still require a power source like a battery or connection to a power socket, proper control and maintenance.

Instead of these active devices, we aimed to propose a passive system that reduces MTC variability during walking. In particular, we considered providing additional sensory input to the ankle tendons, which play important roles in proprioception of the ankle-foot complex [12,13]. One plausible method to enhance motor performance is providing cutaneous stimulation. Light cutaneous inputs to the upper and lower limbs mitigate postural oscillations in standing balance for healthy young adults, the elderly, and diabetic patients [[14], [15], [16]]. Adding tape to skins also enhances joint position accuracy and movement detection by virtue of the additional sensory inflow to the central nervous system (CNS) [17–19]. Although light cutaneous stimulation by touching the skin enhances the motor performance in static tasks like balancing, the efficacy of such intervention in dynamic tasks like walking needs to be investigated.

During dynamic tasks involving ankle movement or sway, wearing orthosis or orthotics enhances ankle proprioception [20,21]. However, such ankle support devices including braces and bandages have non-negligible inertia or restrict the degrees of freedom [22]. In contrast, attaching elastic adhesive tape to skins can effectively produce cutaneous stimulation with minimal inertia and resistance. Strips of tape applied to the ankle tendons enhance proprioception of the ankle joint [19], and cutaneous stimulation through adhesive tape over the skin of the Achilles tendon improve standing balance for healthy young adults and the elderly [[23], [24], [25]]. However, the efficacy of adhesive tape-induced cutaneous stimulation in modifying gait variability has not been explored yet.

In this study, we hypothesized that attaching adhesive tape on the ankle tendons can modify MTC distribution. Considering that regulation of MTC depends on proprioception of both dorsi-flexion and plantar-extension, we assessed the effect of taping-induced cutaneous stimulation to both flexor and extensor tendons on MTC distribution. We additionally investigated the effect of taping on the variability of three-dimensional lower limb angles and activations of two prime shank muscles during swing phase.

## 2 Materials and methods

### 2.1 Participants

Thirteen healthy young adults (9 Males and 4 Females; age: 28.23 ± 5.75 years; height: 170.85 ± 6.57 cm; weight: 66.69 ± 12.59 kg) participated in the study. Consulting a previous study that analyzed the effect of tactile stimulation to the soles on MTC variability [11], we selected the effect size as 0.68, and set the p-value for statistical significance and expected power as 0.05 and 0.95, respectively. Using these inputs, G-power software [26] calculated the sample size as 8. We recruited 13 participants who had no known history of cardiovascular, orthopedic, or neuromuscular disorders. We determined the dominant foot of each participant as the foot that the participant prefers to use when kicking a ball. Every participant was informed and provided written consent regarding all the aspects of the study before participation which was approved by the institutional review board (IRB) of Seoul National University (IRB No. 2004/001-016).

### 2.2 Experimental procedure

We provided the participants with an athletic t-shirt, shorts, and ankle socks, and we asked the participants to bring their own athletic shoes to wear during the experiment. We then estimated the participants’ preferred walking speed (PWS) using the process we adopted in previous studies [11,27]. To recap the process briefly, we estimated the PWS by initially asking the participants to walk on a treadmill at 2.5 km/h and increased the speed by 0.1 km/h per 10 s. We asked the participants to verbally report once they reached the speed that best described their daily walking speed. Then, we further increased the speed by 1.0 km/h and decreased it by 0.1 km/h per 10 s until they again reported the speed that best described their daily walking speed. This process was repeated three times, and the average speed was selected as PWS. We then attached 20 retro-reflective markers on the anatomical landmarks of the dominant and non-dominant legs: the anterior and posterior iliac spinae, greater trochanter, medial and lateral epicondyle, medial and lateral malleolus, first and fifth metatarsal, and heel. The coordinates of the markers were recorded using 10 infra-red cameras (Optitrack Prime^X^ 13, Natural Point, Inc., Oregon, USA) at a sampling frequency of 100 Hz.

We also attached surface electromyography (sEMG) sensors (Avante™ Wireless Systems, Delsys Inc., USA) to record activation levels of the gastrocnemius medialis (GM) and tibialis anterior (TA) of dominant and non-dominant limbs at a sampling frequency of 2000 Hz. The participants then performed walking tasks on a treadmill (Model Gait analysis FDM-TDSL-3i, Zebris Inc®, Germany) at their PWS under four taping conditions: no taping (No), taping the tibialis anterior tendon (Front), taping the Achilles tendon (Rear), and taping both tendons (Both). The initiation of the data acquisition of the infra-red cameras and sEMG sensors was synchronized using a sync box.

**Fig. 1:**
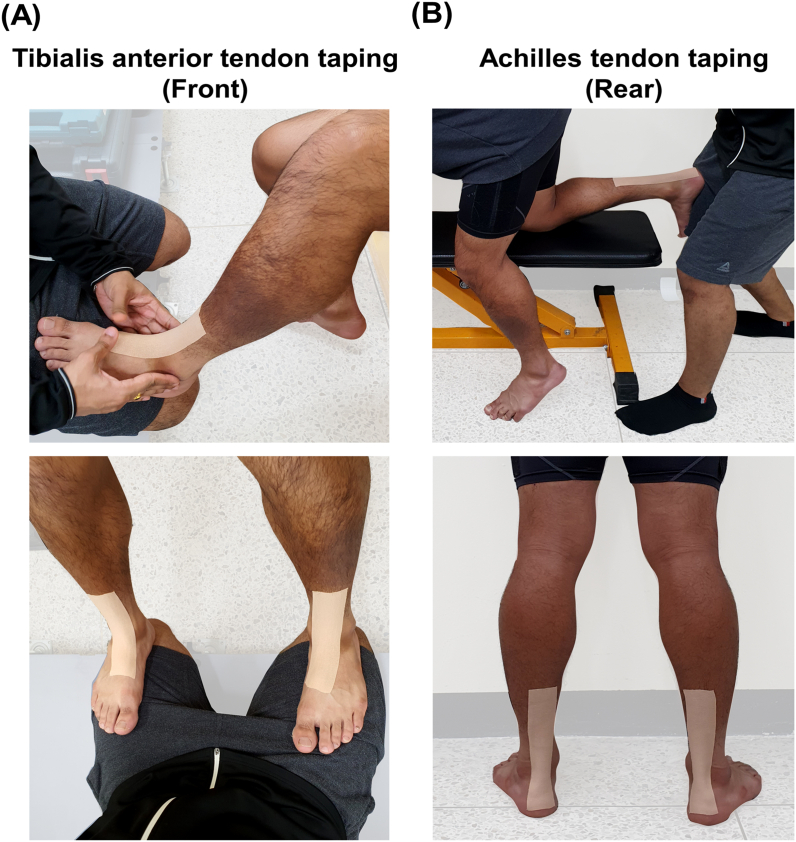
Illustration of the procedure for applying elastic adhesive tape to each tendon. The tape was applied to the (A) tibialis anterior tendons (Front) and (B) Achilles tendons (Rear). Participants were asked to maintain an angle of 90° between the shank and foot. The tape was applied between the insertion points of the tendons.

We used a 5 cm wide elastic adhesive tape (Model No. SX-4113, NIPPON Sigmax Co., Ltd., Japan) to apply cutaneous stimulation to the ankle tendons. Only one investigator applied the tapes for each participant throughout the whole experiment to maintain consistency in the location of the tape application. The process of tape application is illustrated in Fig. 1(A, B). For taping the tibialis anterior tendon (Front), each participant was first asked to sit on a workout bench and place his/her foot on the investigator's thigh, who sat in front of the participant. The investigator ensured that the participant maintained an angle of 90° between the shank and foot. After that, the tape was applied between the distal insertion points of the tibialis anterior muscle. For taping the Achilles tendon (Rear), each participant was asked to bend his/her knee and place it on one of the edges of a workout bench. The participant then stooped forward and placed both hands on the bench while keeping the back parallel to the bench. The investigator then stood behind the participant and placed the participant's foot on the investigator's thigh while ensuring an angle of 90° was maintained between the shank and foot of the participant. After that, the tape was applied between the distal insertion points of the gastrocnemius muscle.

We recorded the position of the retro-reflective markers for 3 s in a standing posture to calibrate the position of the markers before the walking trials. Considering the treadmill acclimatization period [28], we asked participants to walk for 15 min under each condition; without the data during the first 5 min acclimatization period, the remaining 10 min data were used for further processing. To avoid the tape reapplication and maintain the same positions of tape throughout the experiment, the order of taping condition for each participant was pseudo-randomly selected among four sequences: (1) No, Front, Both, and Rear; (2) Front, Both, Rear, and No; (3) No, Rear, Both, and Front; and (4) Rear, Both, Front, and No. For each participant, all the walking trials were performed in a single day, and we provided 10 min rest between trials. We checked the tapes after every trial to confirm that the tape was not peeled off due to excessive sweating. None of the participants sweated to such an extent that the sweat might reduce the adhesion of the tapes.

### 2.3 Data analysis

We used a zero-lag low pass Butterworth filter with a cut-off frequency of 10 Hz to filter the raw coordinates of the retro-reflective makers. Using biomechanical modeling software (Visual3D v6™, C-Motion, Inc., Maryland, USA), we built a seven-segmental model with a pelvis, thighs, shanks, and feet to calculate lower limb joint kinematics.

#### 2.3.1 Minimum toe clearance (MTC)

We used the proximal-distal trajectory of the retro-reflective marker attached to the first metatarsal head (Meta-1_Z_) to detect MTC. First, we averaged the Meta-1_Z_ recorded during the 3 s of standing, which was set as the ground position of the toe. We subtracted this ground position of the toe from the Meta-1_Z_ trajectory during the 10 min of the data acquisition period. To define MTC, we defined gait cycles. We defined one gait cycle from a heel strike (HS) of one foot to the successive HS of the same foot. Consulting a previous study [29], the moment of HS was identified as the time point of the local maximum of the distance between the anterior-posterior position of the pelvis center of mass and the heel marker. We extracted 492 to 572 gait cycles during the 10 min data acquisition period. For each gait cycle, the Meta-1_Znorm_ trajectory showed two local maxima and one local minimum between the maxima, and MTC was defined as the local minimum of Meta-1_Z_. The MTC height and variability of Meta-1_Z_ were defined as the average and standard deviation (SD) of the local minimum height of all the strides during the 10 min of walking.

#### 2.3.2 Lower limb joint angles

Visual 3D software was used to calculate the three-dimensional ankle, knee, and hip angles. Following the default Cardan sequence of the software, the coordinate system was defined as flexion/extension, abduction/adduction, and axial rotation, which denotes the joint movements in sagittal, frontal, and transverse planes, respectively. We then extracted the three-dimensional lower limb joint angles at the time point of MTC. Then, the average and standard deviation of the three-dimensional joint angles during the 10 min of walking data acquisition period were extracted under each taping condition. We performed correlation analysis to investigate the association between the variability of three-dimensional joint angles and MTC.

#### 2.3.3 Integrated electromyography (IEMG)

We calculated the activation of the gastrocnemius medialis (GM) and tibialis anterior (TA) during swing phase of each limb. The swing phase was defined as the time period from toe-off (TO) to heel strike (HS). Consulting a previous study [29], we selected TO as the time point of the local minimum of the distance between the anterior-posterior position of the pelvis center of mass and the marker attached at the first metatarsal head. After extracting the raw EMG signals during each swing phase, we filtered the signals using a zero-lag fourth-order low-pass Butterworth filter with cut-off frequencies between 20 Hz and 350 Hz. We then rectified the filtered EMG signals and further smoothened the signals using a moving average zero-lag filter with a time window equal to 1% of the length of the EMG signals during each step. We then calculated the integrated EMG (IEMG) for each step using Eq. (1):

(1) $$IEMG=\int_{HS}^{TO}EMG\;dt$$

We calculated the maximum IEMG (IEMG_Max_) following the method outlined in a previous study [30]. We stacked all the moving average filtered EMG data during swing phase and calculated the IEMG values for a time window corresponding to 1% of the length of the stacked dataset of all the steps for all the taping conditions. IEMG_Max_ was selected as the maximum IEMG value within these time windows. Then, to calculate the normalized IEMG or IEMG_Norm_, we divided the IEMG by the IEMG_Max_ as in Eq. (2) across all the steps for the four taping conditions:

(2) $${IEMG}_{Norm}=\frac{IEMG}{{IEMG}_{\mathrm{Max}}}$$

Finally, we calculated the average of the IEMG_Norm_ for GM and TA of both limbs for 10 min of walking under each taping condition.

### 2.4 Statistical analysis

We performed one-way repeated measures analysis of variance (ANOVA) to assess the differences in the MTC height, MTC variability, three-dimensional lower limb joint angles, and IEMG_Norm_ of GM and TA for 13 participants depending on the taping conditions (No, Front, Rear, and Both) separately for the dominant and non-dominant limb. We used Bonferroni correction as the post-hoc test for multiple pairwise comparisons. Greenhouse-Geisser criterion was used to reduce the degrees of freedom in case that the assumption of sphericity was violated according to Mauchly's test. Pearson's correlation analysis was performed to assess the strength of the linear relationship between MTC variability and joint angle variability. The level of statistical significance was set at p < 0.05.

## 3 Results

### 3.1 Minimum toe clearance (MTC)

**Fig. 2:**
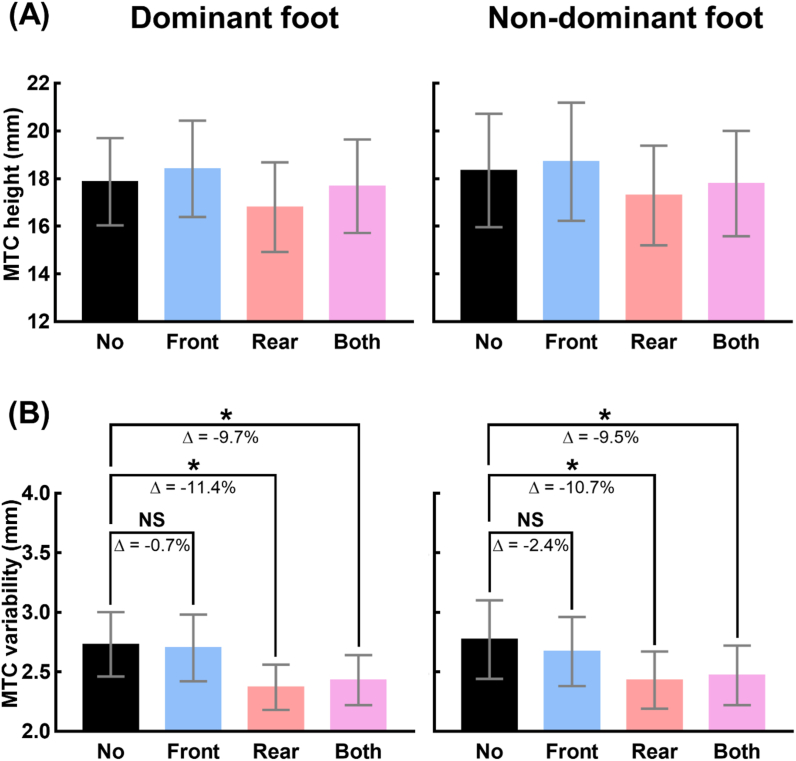
Changes in the MTC distribution due to application of tape to the ankle tendons. (A) and (B) show the means and standard errors of the values of MTC height and variability of 13 participants, respectively, under the four taping conditions (No: no taping, Front: taping the tibialis anterior tendon, Rear: taping the Achilles tendon, Both: taping both tendons). The asterisk indicates statistically significant difference (*: p < 0.05). The reduction in variability is quantified as Δ (%), the ratio of the difference to the values under No condition.

The mean and standard error of the MTC height and its variability of 13 participants under the four taping conditions for both feet are shown in Fig. 2(A, B). The results of one-way repeated measures ANOVA revealed a significant main effect of taping condition on the values of MTC height for both feet (dominant: F_[3,36]_ = 3.138, p = 0.037, *η*^2^ = 0.207; non-dominant: F_[3,36]_ = 2.970, p = 0.045, *η*^2^ = 0.198). However, pairwise comparisons revealed no significant differences in the values of MTC height between taping conditions.

A significant main effect of taping conditions was observed on the values of MTC variability of 13 participants for both feet (dominant: F_[1.710,20.519]_ = 6.808, p = 0.007, *η*^2^ = 0.362; non-dominant: F_[2.061,24.732]_ = 7.305, p = 0.003, *η*^2^ = 0.378). For both feet, the results of pairwise comparisons revealed that MTC variability decreased significantly under Rear (dominant: p = 0.019, non-dominant: p = 0.022) and Both (dominant: p = 0.014, non-dominant: p = 0.036) conditions compared to No condition. The percentage difference in MTC variability under each of Front, Rear, and Both conditions with respect to the variability under No condition for 13 participants was also quantified as Δ (%) in Fig. 2(A, B). For both feet, the average Δ values were above 9% under Rear and Both conditions, whereas it was below 3% under Front condition.

### 3.2 Lower limb joint angles

**Fig. 3:**
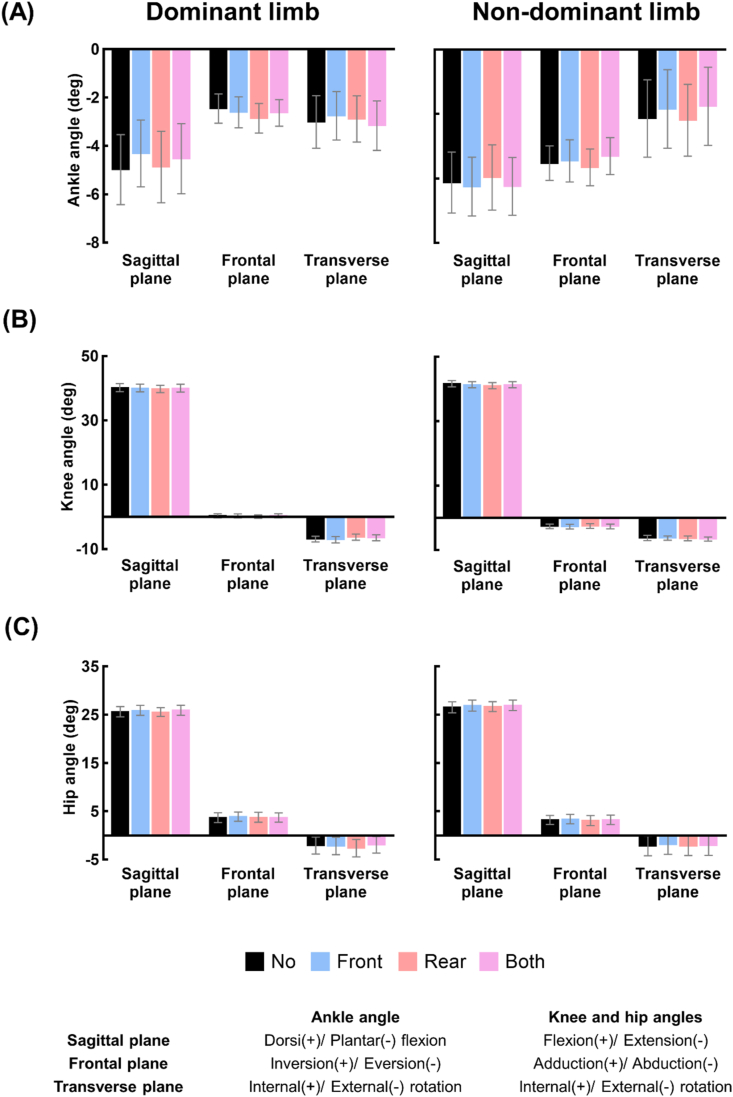
Changes in the lower limb joint angles at the time points of MTC due to application of tape to the ankle tendons. (A)–(C) show the means and standard errors of the values of the ankle, knee, and hip angles at the time points of MTC of 13 participants, respectively, under the four taping conditions (No: no taping, Front: taping the tibialis anterior tendon, Rear: taping the Achilles tendon, Both: taping both tendons).

**Fig. 4:**
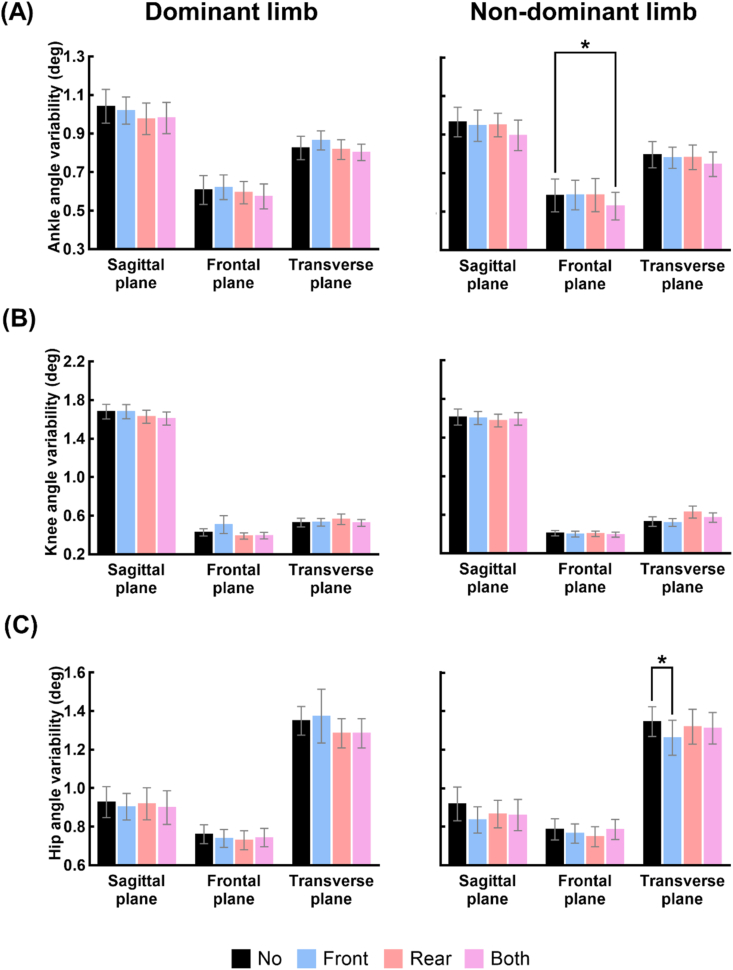
Changes in the variability of lower limb joint angles at the time points of MTC due to application of tape to the ankle tendons. (A)–(C) show the means and standard errors of the variability of the ankle, knee, and hip angles at the time points of MTC of 13 participants, respectively, under the four taping conditions (No: no taping, Front: taping the tibialis anterior tendon, Rear: taping the Achilles tendon, Both: taping both tendons). The asterisk indicates statistically significant difference (*: p < 0.05).

Fig. 3(A–C) and 4(A–C) show the mean and standard error of the lower limb joint angles and variabilities under the four taping conditions. Supplementary Table 1 shows the results of the one-way repeated measures ANOVA by which we assessed the effect of the taping condition on the lower limb joint angles. Significant main effects of taping condition on the knee angles in the transverse and sagittal planes were observed for the dominant and non-dominant limbs, respectively, whereas pairwise comparisons revealed no significant differences in the three-dimensional lower limb joint angles between taping conditions.

Supplementary Table 2 shows the results of the one-way repeated measures ANOVA by which we assessed the effect of the taping condition on the variability of the lower limb joint angles. Significant main effects of taping condition on the ankle angle variability in the frontal plane, and hip angle variability in the sagittal and transverse plane were observed for the non-dominant limb. Pairwise comparisons revealed that the ankle angle variability in the frontal plane under Both condition and the hip angle variability in the transverse plane under Front condition were significantly lower than the variability under No condition for non-dominant limb.

### 3.3 Association between MTC and lower limb joint angles variability

The coefficient of determination (*R*^*2*^) and correlation coefficient (*r*) values from Pearson's correlation analysis are compiled in Table 1. Significant positive correlations were observed between MTC variability and joint angle variability in all three planes except knee angle variability in the frontal plane. *R*^*2*^ and *r* values for the sagittal, frontal, and transverse planes were largest for the ankle, hip, and knee joints, respectively. *R*^*2*^ and *r* values for the sagittal plane were always higher than those for frontal and transverse planes.

**Table 1:** The results of the Pearson's correlation analysis between minimum toe clearance (MTC) variability and the variability of lower limb joint angles.

Joint	Sagittal plane	Frontal plane	Transverse plane
Ankle	*R*^*2*^ = 0.525	*R*^*2*^ = 0.150	*R*^*2*^ = 0.145
	*r* = 0.728	*r* = 0.398	*r* = 0.392
	p < 0.001	p < 0.001	p < 0.001
Knee	*R*^*2*^ = 0.334	*R*^*2*^ = 0.026	*R*^*2*^ = 0.146
	*r* = 0.583	*r* = 0.187	*r* = 0.393
	p < 0.001	p = 0.057	p < 0.001
Hip	*R*^*2*^ = 0.318	*R*^*2*^ = 0.243	*R*^*2*^ = 0.126
	*r* = 0.569	*r* = 0.500	*r* = 0.367
	p < 0.001	p < 0.001	p = 0.001

*R*^*2*^ and *r* represent the coefficient of determination and correlation coefficient, respectively.

### 3.4 Normalized integrated EMG (IEMG_Norm_)

**Fig. 5:**
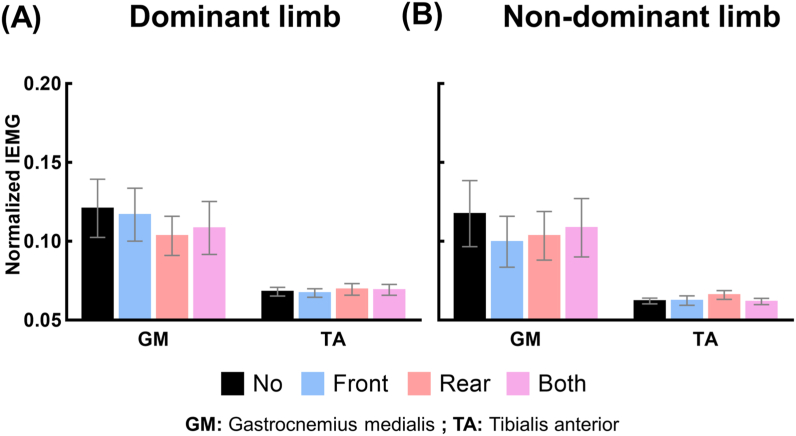
Changes in the activation level of shank muscles. (A) and (B) show the values of normalized integrated EMG of two muscles (GM: Gastrocnemius medialis and TA: Tibialis anterior) during swing phase under the four taping conditions (No: no taping, Front: taping the tibialis anterior tendon, Rear: taping the Achilles tendon, Both: taping both tendons) for the dominant and non-dominant limb, respectively.

The mean and standard error of IEMG_Norm_ of GM and TA of both limbs during swing phase under the four taping conditions are shown in Fig. 5(A, B). Supplementary Table 3 shows the results of the one-way repeated measures ANOVA by which we assessed the effect of the taping condition on the IEMG_Norm_ of the GM and TA of both limbs during swing phase. No significant main effect of taping conditions was revealed on IEMG_Norm_ of these muscles.

## 4 Discussion

Our results indicate that practical, simple, and minimally interfering intervention of applying elastic adhesive tape to the ankle tendons can effectively reduce MTC variability. We found that applying tape to the Achilles tendons significantly reduced MTC variability, whereas applying tape only to the tibialis anterior tendons does not make significant changes. We also found that tape applied to the Achilles tendons reduced the variability of some lower limb joint angles.

The rationale for using tape was to induce proper cutaneous stimulation, which increases the inflow of sensory input to the CNS to better localize the joint movement [31]. This, in turn, enables the CNS to improve the accuracy of detecting the joint position and movement [17,18]. Simoneau et al. showed that tape applied to the ankle tendons reduces the position error of the ankle joint and improves proprioception in the sagittal plane [19]. Hence, the observed decrease in MTC variability was plausibly due to the tape-induced additional proprioceptive inflow to the CNS. The tape-induced decline in joint angle variabilities in some planes is also consistent with the enhanced proprioception. The decreased variability can also be partly attributed to the enhanced neural coherence at the somatosensory cortex, which is known to be evoked by sensory stimulation to the Achilles tendon [32].

Table 1 shows that the largest proportion of the variance of MTC variabilities is explained by changes in the sagittal plane ankle angle variabilities. This observation is consistent with the results of previous studies which demonstrated that the regulation of the sagittal plane ankle angle dominantly contributes to the modified MTC distribution [33,34]. However, in contrast with the result of a previous study by Perera et al., which reported a concurrence of an increase in the activation of the shank muscles and a decrease in MTC variability [33], we did not observe any significant effect of the taping intervention on the muscle activations despite the clear decrease in MTC variability due to taping. Our results suggest that an increase in the activation of shank muscles is not a necessary condition for the reduction in the MTC variability.

Interestingly, the significant decline in MTC variability and joint angle variabilities was elicited only when the tape was applied to the Achilles tendons or both tendons; the tape applied to the tibialis anterior alone could not induce a significant change in MTC distribution. Considering that the Achilles tendon is the largest tendinous structure in the human body [35,36], it is reasonable to assume that a large number of sensory receptors are embedded in this tendon. This might also partly explain the efficacy of tape applied to the Achilles tendons in improving balance [23–25]. In contrast, the number of sensory receptors in the tibialis anterior tendons might not be large enough to significantly add the inflow of sensory input to the CNS when the tendons are stimulated only by the elastic tape.

The decreased MTC variability may also be related with enhanced gait automaticity. During steady-state walking, CNS expends attention-demanding executive resources for reliable MTC control, and reducing the use of these resources leads to higher gait automaticity [37]. Previous studies used dual-task walking to divert executive resources away from walking and explore its effect on gait automaticity and MTC distribution [38,39]. Hamacher et al. found that dual-task walking decreased MTC variability by 11.3% for young adults and 24.3% for the elderly [40]. These studies postulated that the distracting task minimized executive resources for MTC control during walking and shifted control of MTC towards sub-cortical and spinal levels. The shift in strategy decreased MTC variability by increasing gait automaticity, resulting in a more reliable control of MTC. By the way, other previous studies have shown that cutaneous stimulation to the soles of feet reduces cortical activation during walking, and the cutaneous input to the skin can regulate spinal motor neuronal activity [41,42]. Although we did not directly record the cortical activity, these results of multiple previous studies suggest that cutaneous stimulation applied to the skin of ankle tendons might contribute to enhancing gait automaticity and accordingly reducing MTC variability.

Our study has several limitations that needs to be clarified. First, we recruited only young and healthy adults. The efficacy of the same intervention of taping in the elderly and patients who typically exhibit larger MTC variability due to sensorimotor dysfunctions needs to be assessed in a future study. However, aging and neuromuscular diseases result in a decline in muscle strength. Therefore, the possible effect of taping on muscle weakness also needs to be considered before suggesting taping as an intervention to reduce MTC variability. Second, we asked the participants to walk on a treadmill to obtain a sufficient number of gait cycles and perform valid analyses. However, there are subtle differences between treadmill and overground walking [43], so the efficacy of the suggested intervention in reducing MTC variability during daily overground walking needs to be clarified by additional studies. Finally, we assessed the acute effect of taping on MTC distribution, which limits the insight on the translational applicability of our results. Future studies are required to investigate any possible long-term effect of taping on MTC distribution.

## Author contribution statement

Prabhat Pathak: conceived and designed the experiments; performed the experiments; analyzed and interpreted the data; contributed to materials, analysis tools or data; and wrote the paper.

Jooeun Ahn: contributed to materials, analysis tools or data; and wrote the paper.

## Funding statement

This work was supported in part by the Technology Innovation Program (No. 20008912), Industrial Technology Innovation Program (No. 20007058, Development of safe and comfortable human augmentation hybrid robot suit), and Industrial Strategic Technology (No. 20018157) funded by the Ministry of Trade, Industry & Energy (MOTIE, Korea), and 10.13039/501100003725National Research Foundation of Korea (NRF) Grants funded by the Korean Government (MSIT) (No. 2016R1A5A1938472).

## Data availability statement

All data sets generated and/or analyzed during the current study are available from the corresponding author (Jooeun Ahn) on reasonable request.

## Declaration of competing interest

The authors declare no conflict of interest. The funding organizations had no role in the study design, in the collection, analysis and interpretation of data; in the writing of the manuscript; and in the decision to submit the manuscript for publication.
