# Supplementary material for: Taping-induced cutaneous stimulation to the ankle tendons reduces minimum toe clearance variability
Source: Heliyon. 2023 Jan 6;9(1):e12682. doi: 10.1016/j.heliyon.2022.e12682 (PMC9850051; doi:10.1016/j.heliyon.2022.e12682)
Supplement: Multimedia component 1 [file mmc1.docx]

**SUPPLEMENTARY MATERIALS**

**Supplementary Table 1.** The results of one-way repeated measures ANOVA: effects of taping

conditions on lower limb joint angles

| **Joint** | | **Within-subject effects** | | |
| --- | --- | --- | --- | --- |
|  |  | **Sagittal plane** | **Frontal plane** | **Transverse plane** |
| **Ankle** | **Dominant** | F_[3,36]_ = 1.766,  p = 0.171,  *η^2^* = 0.128 | F_[3,36]_ = 1.025,  p = 0.393,  *η^2^* = 0.079 | F_[3,36]_ = 0.795,  p = 0.505,  *η^2^* = 0.062 |
|  | **Non-dominant** | F_[1.81,21.68]_ = 0.392,  p = 0.660,  *η^2^* = 0.032 | F_[3,36]_ = 0.906,  p = 0.448,  *η^2^* = 0.070 | F_[3,36]_ = 1.631,  p = 0.199,  *η^2^* = 0.120 |
| **Knee** | **Dominant** | F_[1.66,19.92]_ = 1.701,  p = 0.210,  *η^2^* = 0.124 | F_[1.29,15.53]_ = 1.579,  p = 0.234,  *η^2^* = 0.116 | **F_[3,36]_ = 3.297,**  **p = 0.031,**  ***η^2^* = 0.216** |
|  | **Non-dominant** | **F_[3,36]_ = 4.006,**  **p = 0.015,**  ***η^2^* = 0.250** | F_[1.61,19.35]_ = 1.088,  p = 0.344,  *η^2^* = 0.083 | F_[3,36]_ = 0.205,  p = 0.892,  *η^2^* = 0.017 |
| **Hip** | **Dominant** | F_[3,36]_ = 0.846,  p = 0.478,  *η^2^* = 0.066 | F_[3,36]_ = 0.888,  p = 0.457,  *η^2^* = 0.069 | F_[1.41,16.95]_ = 1.768,  p = 0.204,  *η^2^* = 0.128 |
|  | **Non-dominant** | F_[3,36]_ = 1.362,  p = 0.270,  *η^2^* = 0.102 | F_[3,36]_ = 1.589,  p = 0.209,  *η^2^* = 0.117 | F_[3,36]_ = 0.613,  p = 0.611,  *η^2^* = 0.049 |

**Supplementary Table 2.** The results of one-way repeated measures ANOVA: effects of taping conditions on the variability of lower limb joint angles

| **Joint** | | **Within-subject effects** | | |
| --- | --- | --- | --- | --- |
|  |  | **Sagittal plane** | **Frontal plane** | **Transverse plane** |
| **Ankle** | **Dominant** | F_[3,36]_ = 0.834,  p = 0.484,  *η^2^* = 0.065 | F_[3,36]_ = 0.734,  p = 0.539,  *η^2^* = 0.058 | F_[3,36]_ = 0.882,  p = 0.459,  *η^2^* = 0.068 |
|  | **Non-dominant** | F_[3,36]_ = 1.965,  p = 0.269,  *η^2^* = 0.102 | **F_[3,36]_ = 3.888,**  **p = 0.017,**  ***η^2^* = 0.245** | F_[3,36]_ = 1.370,  p = 0.268,  *η^2^* = 0.102 |
| **Knee** | **Dominant** | F_[3,36]_ = 1.800,  p = 0.165,  *η^2^* = 0.130 | F_[1.04,12.48]_ = 1.396,  p = 0.261,  *η^2^* = 0.104 | F_[1.55,18.59]_ = 0.508,  p = 0.564,  *η^2^* = 0.041 |
|  | **Non-dominant** | F_[3,36]_ = 0.448,  p = 0.720,  *η^2^* = 0.036 | F_[3,36]_ = 0.535,  p = 0.661,  *η^2^* = 0.043 | F_[1.427,17.127]_ = 2.502,  p = 0.123,  *η^2^* = 0.173 |
| **Hip** | **Dominant** | F_[3,36]_ = 0.170,  p = 0.916,  *η^2^* = 0.014 | F_[3,36]_ = 0.574,  p = 0.636,  *η^2^* = 0.046 | F_[1.31,15.71]_ = 0.510,  p = 0.534,  *η^2^* = 0.041 |
|  | **Non-dominant** | **F_[3,36]_ = 3.376,**  **p = 0.029,**  ***η^2^* = 0.220** | F_[3,36]_ = 1.778,  p = 0.169,  *η^2^* = 0.129 | **F_[3,36]_ = 3.537,**  **p = 0.024,**  ***η^2^* = 0.228** |

**Supplementary Table 3.** The results of one-way repeated measures ANOVA: effects of taping conditions on the normalized integrated electromyography (IEMG_Norm_) of the gastrocnemius medialis (GM) and tibialis anterior (TA) muscles

|  | **Within-subject effects** | |
| --- | --- | --- |
|  | **Gastrocnemius medialis (GM)** | **Tibialis anterior (TA)** |
| **Dominant** | F_[1.500,18.003]_ = 0.980,  p = 0.371,  *η^2^* = 0.076 | F_[3,36]_ = 0.655,  p = 0.585,  *η^2^* = 0.052 |
| **Non-dominant** | F_[1.460,17.523]_ = 0.900,  p = 0.394,  *η^2^* = 0.070 | F_[1.784,21.409]_ = 2.212,  p = 0.138,  *η^2^* = 0.156 |
